# Supplementary material for: Exploring the Effects of Different Drying Methods on Related Differential Metabolites of Pleurotus citrinopileatus Singer Based on Untargeted Metabolomics
Source: Plants (Basel). 2024 Jun 7;13(12):1594. doi: 10.3390/plants13121594 (PMC11207783; doi:10.3390/plants13121594)
Supplement: Supplementary file 1 [file plants-13-01594-s001.zip › plants-2978366-supplementary/Supplementary figures-plants20240408.pdf]

Supplementary Material

1 Supplementary Figures

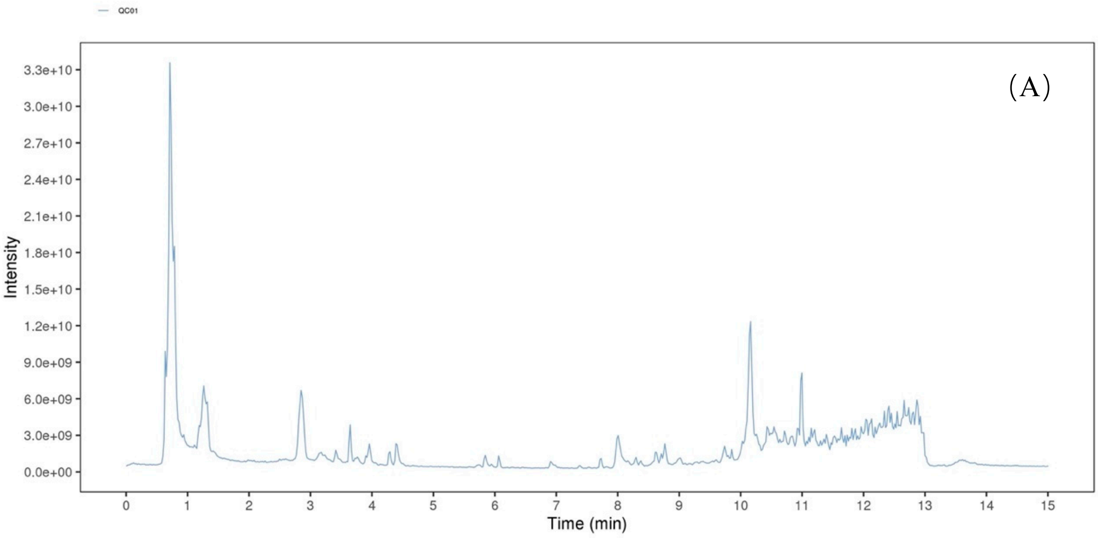

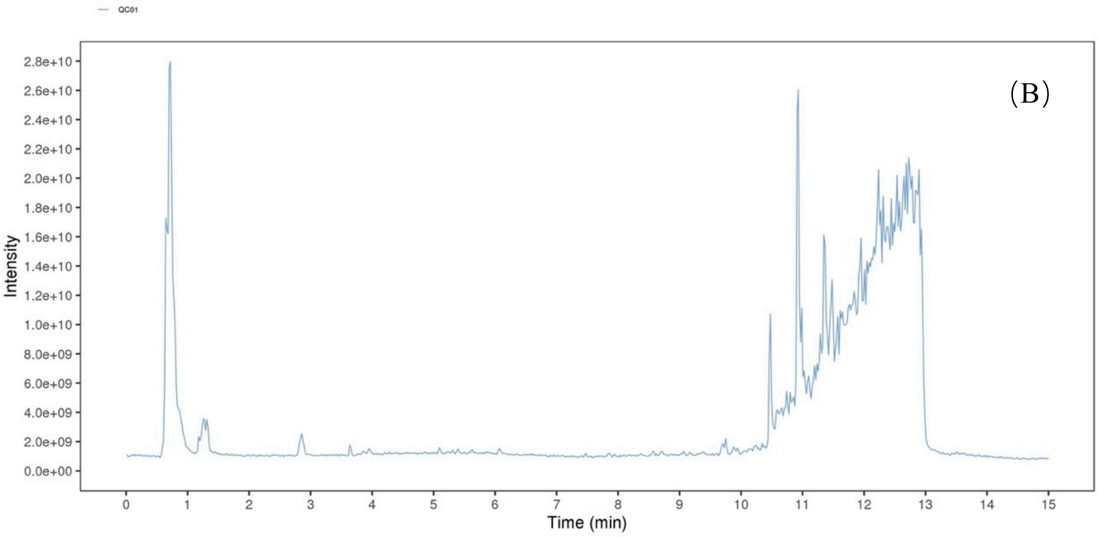

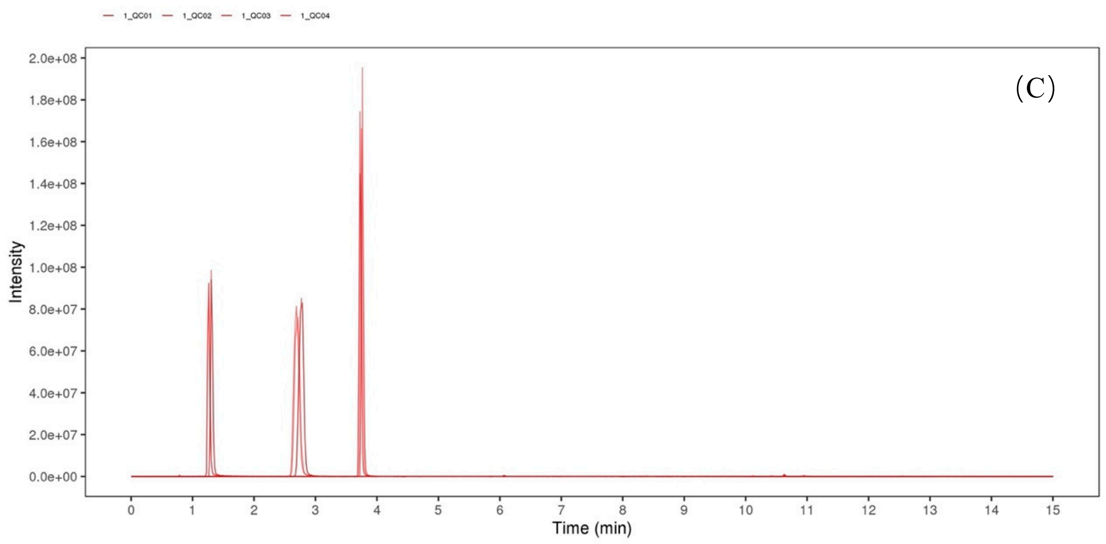

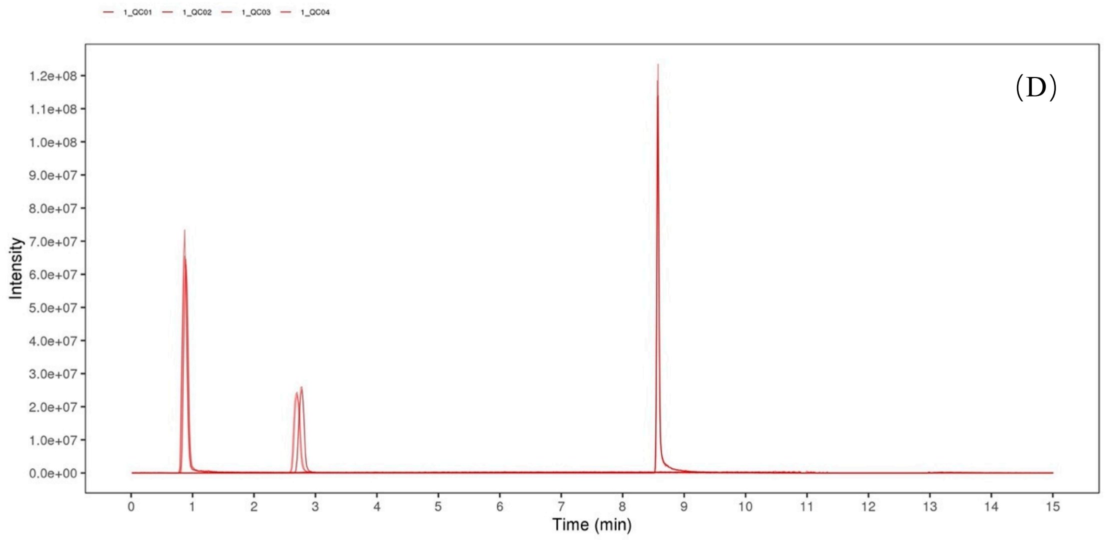

Figure S1. Total ion current (TIC) diagram of the quality control (QC) samples in (**A**) electrospray ionization (ESI)+ and (**B**) in ESI– mode. The extract ion chromatogram (EIC) of the internal standard in the QC sample in positive (**C**) and negative ion modes (**D**).

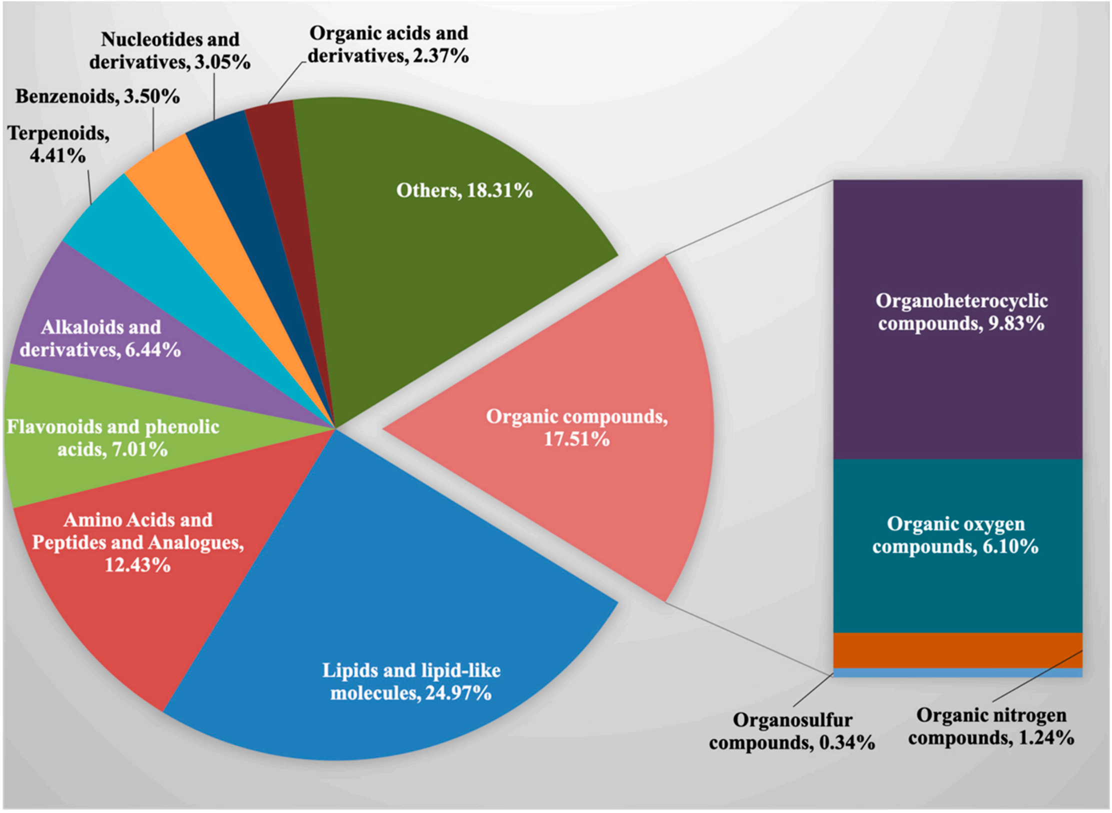

Figure S2. Metabolite classes and quantities detected in the PCS samples of the three drying methods. The pie chart representing the percentage of metabolites belong to superclass of metabolites.

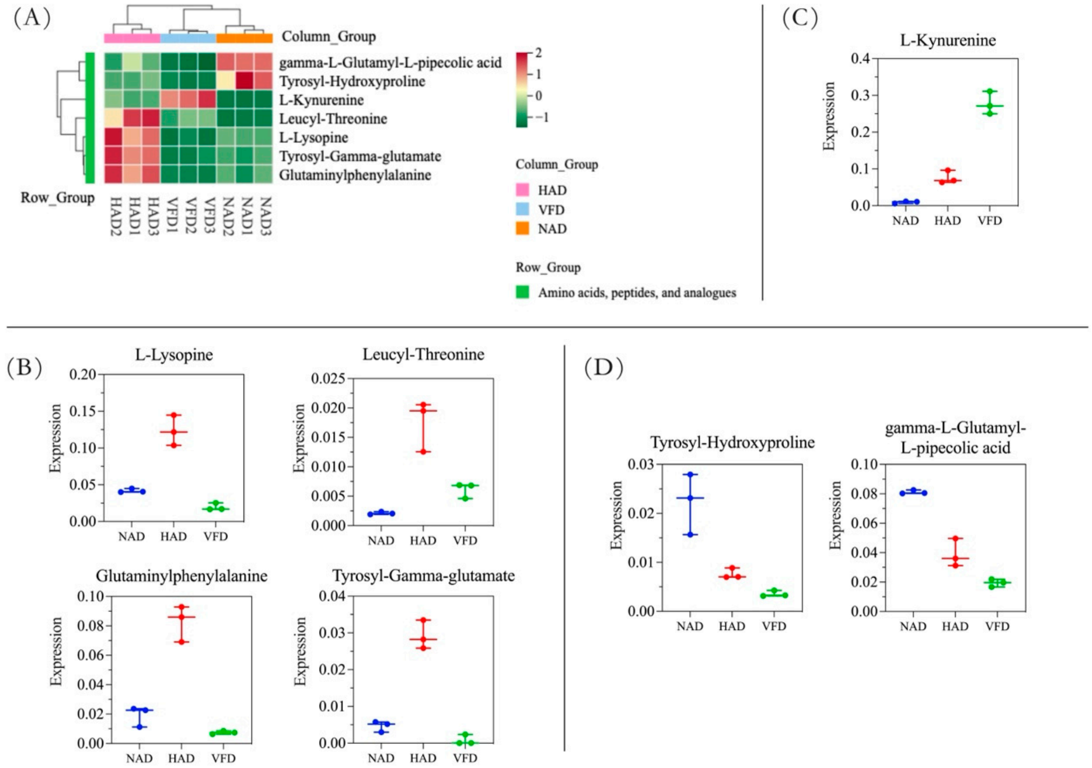

Figure S3. Heatmap showing the accumulation pattern of amino acids, peptides, and analogues among PCs obtained from 3 drying methods. Boxplots showing the relative abundance of 7 differential DMs of amino acids, peptides, and analogues (Data are expressed as mean ± SD, y-axis represents the value of relative quantitative level）.

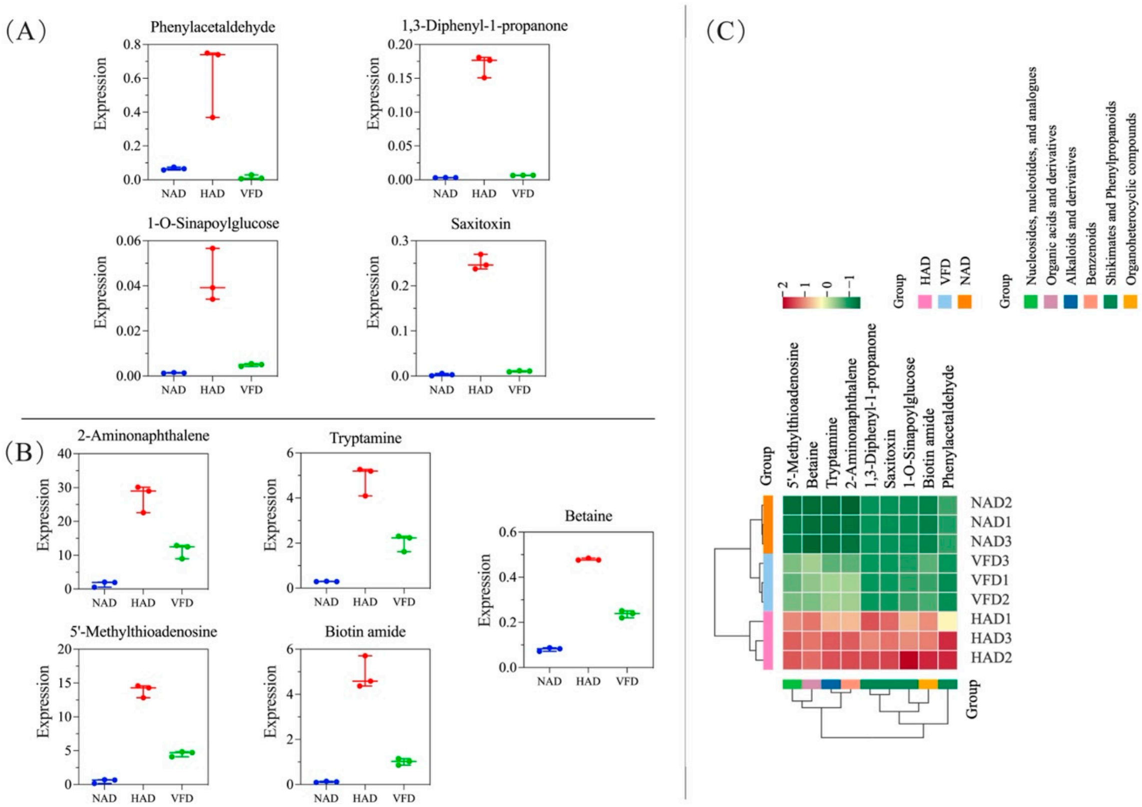

Figure S4. Heatmap showing the accumulation pattern of other DMs among PCs obtained from 3 drying methods. Boxplots showing the relative abundance of other 9 differential DMs of PCS. (Data are expressed as mean ± SD, y-axis represents the value of relative quantitative level)
